# Evaluation of surgery and surgical results of Baha® Attract system implantations – single centre experience of hundred twenty five cases^☆^

**DOI:** 10.1016/j.bjorl.2018.04.011

**Published:** 2018-05-31

**Authors:** Wojciech Gawęcki, Andrzej Balcerowiak, Ewelina Kalinowicz, Maciej Wróbel

**Affiliations:** Poznań University of Medical Sciences, Department of Otolaryngology and Laryngological Oncology, Poznań, Poland

**Keywords:** Bone conduction, Hearing aids, Hearing loss, Condução óssea, Aparelhos auditivos, Perda auditiva

## Abstract

**Introduction:**

Bone-anchored hearing aids are currently well-established solutions for treatment of hearing-impaired patients.

**Objective:**

To evaluate the surgery of the Baha® Attract system, healing process and soft tissue condition after the processor activation.

**Methods:**

125 patients implanted with the Baha® Attract system during a 3 year period in a single ENT department were analysed. Evaluated parameters comprised: details of surgery, healing process and soft tissue condition at the time of the processor activation and on subsequent follow-up visits.

**Results:**

The implantation was conducted under local anaesthesia in 96% of patients. The mean surgery time was 42 min. Soft tissue reduction was performed in 43.2% of cases; bone polishing in 23.2% and bipolar coagulation in all the cases. Healing was uneventful in 92.8%. 10 days after the surgery, pain was reported in 48% of cases. On subsequent follow-up visits, 1 month and 3 months after the surgery, pain was present in 18.4% and 2.4% of cases respectively. Similarly, numbness and paresthesia, initially reported in 84% and 15.2%, were present in 60% and 11.2% after a month, and in 17.6% and 1.6% after three months. After the processor attachment, no serious problems were observed in the analysed group during follow-up visits. However, mild redness and/or mild pain over the magnet were observed in 9.6% of patients.

**Conclusion:**

Implantation of the Baha® Attract system is an easy and safe procedure. It can be performed under local anaesthesia in adults. There are no major surgical problems or complications, and the healing process proceeds efficiently in most patients. Postoperative pain is usually mild and gradually decreases in the following months. Numbness in the operated area is frequent, but as reinnervation occurs in time, the numb patch decreases in size and finally completely disappears in most cases.

## Resumo

**Introdução:**

Os processadores de implantes auditivos de ancoragem óssea são atualmente soluções bem estabelecidas para o tratamento de pacientes com deficiência auditiva.

**Objetivo:**

Avaliar a cirurgia de implante do sistema Baha® Attract, o processo de cicatrização e a condição dos tecidos moles após a ativação do processador.

**Método:**

Foram analisados 125 pacientes implantados com o sistema Baha® Attract durante um período de 3 anos em um único departamento de otorrinolaringologia. Os parâmetros avaliados compreenderam: detalhes da cirurgia, processo de cicatrização e condição dos tecidos moles no momento da ativação do processador e nas consultas de seguimento subsequentes.

**Resultados:**

O implante foi realizado sob anestesia local em 96% dos pacientes. O tempo médio de cirurgia foi de 42 minutos. A redução de tecido mole foi realizada em 43,2% dos casos; polimento ósseo em 23,2% e coagulação bipolar em todos os casos. A cicatrização transcorreu sem complicações em 92,8%. Dez dias após a cirurgia, dor foi relatada em 48% dos casos. Nas consultas de seguimento subsequentes, 1 mês e 3 meses após a cirurgia, a dor esteve presente em 18,4% e 2,4% dos casos, respectivamente. Da mesma forma, dormência e parestesia, inicialmente relatados em 84% e 15,2%, estavam presentes em 60% e 11,2% dos casos após um mês, e em 17,6% e 1,6% após três meses. Após a fixação do processador, nenhum problema grave foi observado no grupo analisado durante as consultas de seguimento. No entanto, vermelhidão leve e/ou dor leve sobre o ímã foram observados em 9,6% dos pacientes.

**Conclusão:**

O implante do sistema Baha® Attract é um procedimento fácil e seguro. Ele pode ser realizado sob anestesia local em adultos. Não há grandes problemas ou complicações cirúrgicas, e o processo de cicatrização é contínuo e eficaz na maioria dos pacientes. No pós-operatório, a dor é geralmente leve e diminui gradualmente nos meses seguintes. A dormência na área operada é freqüente, mas como a reinervação ocorre com o tempo, a área dormente diminui de tamanho e finalmente desaparece por completo na maioria dos casos.

## Introduction

Bone-anchored hearing aids (BAHAs) or bone conduction hearing devices (BCHDs) are currently well-established solutions for treatment of hearing-impaired patients. They are used in patients with unilateral and bilateral, mixed and conductive hearing loss as well as with single-sided deafness. The first implantation was reported by Tjellström and Granström in 1977,^1^ and since then, more than 150,000 individuals have been implanted worldwide.^2^ Nowadays, two types of bone conduction implants are available for patients: percutaneous (skin penetrating) and transcutaneous (non-skin penetrating).

The percutaneous BAHA is composed of a titanium implant connected with a percutaneous abutment and a sound processor, which is attached to the abutment. Such solution enables direct and high quality sound transmission, but it requires lifelong daily hygienic care. There is also risk of local skin complications including infections, skin overgrowth and sometimes even implant loss.^3^, ^4^ The frequency of recurrent soft tissue reactions and infections around the abutment was reported in 8–59% of cases, implant loss in 8.3%, and the need for revision surgery in 5–42%.^3^ Additionally, the cosmetic effect is not optimal, and some patients, who could benefit from the system, decline.^5^ Nowadays, there are two percutaneous systems commercially available: Baha® Connect (Cochlear Ltd, Australia) and Ponto® (Oticon A/S, Denmark).

The transcutaneous BAHA uses magnetic attraction force to attach the processor over the implant instead of the abutment. There is no permanent skin defect, hence no hygienic problems. Aesthetic effect is also good. Presently, three different systems are available on the market: Sophono® (Medtronic, USA), Bonebridge® (Medel, Austria) and Baha® Attract (Cochlear Ltd, Australia).

The system reported in the study – Baha® Attract (Cochlear Bone Anchored Solutions AB, Mölnlycke, Sweden) – was introduced in 2013. It is composed of a titanium implant, a sound processor and two magnetic discs: one under the skin, which is connected to the implant, and the second one – external, to which the sound processor is attached. Additionally, a pad of soft material covers the surface of the external magnet and distributes the pressure to the skin and soft tissue between the magnets.

The aim of the study is to evaluate the surgery of the Baha® Attract system, healing process and soft tissue condition after the processor activation.

## Methods

331 patients were implanted with different available systems of bone-anchored solutions in our department between 1992 and September 2017. 125 patients (37.8%) were implanted with the Baha® Attract (Cochlear Bone Anchored Solutions AB, Mölnlycke, Sweden) between September 2014 and September 2017, and all of them were enrolled in this prospective study. The first 20 patients implanted with Baha® Attract in our department, initially reported in previous study,^6^ were also included into analysed group. The surgical procedure was standardised (C-shaped incision consistent with Cochlear Baha® Attract Surgical Quick Guide), proceeded in the same way for every patient, and was performed by the two surgeons (WG and AB) in the same conditions. The clinical chart covering surgical and follow-up aspects of each operated patient enabled to collect data, used for further evaluation. Full data from the study cohort is available. The investigation was approved by the local Ethics Committee (decision no. 1234/17).

### Group characteristics

The whole group of 125 patients (79 females and 46 males, 12–86 y/o with a mean of 52) had no history of conditions that could jeopardise osseointegration and wound healing. The most frequent otological indications for the surgery were chronic otitis media, after unsuccessful trial of sound transmission system reconstruction (54%), and otosclerosis, after unsuccessful stapedotomy or restapedotomy (19%). The most frequent audiological indications were bilateral mixed hearing loss (48%) and single-sided deafness (31%). The details are presented in Table 1.

**Table 1:** Characteristics of the patients (*n* = 125).

Category	*n*	%
*Age*		
<20 y.o. (teenagers)	3	2.4
20–40 y.o.	26	20.8
41–60 y.o.	50	40
61–80 y.o.	45	36
>80 y.o.	1	0.8

*Sex*		
Male	46	36.8
Female	79	63.2

*Otological indications*		
Chronic otitis media	67	53.6
Otosclerosis	24	19.2
Congenital malformation	8	6.4
Acquired atresia of external auditory canal	5	4
Single-sided deafness of unknown aetiology	21	16.8

*Audiological indications*		
Bilateral conductive hearing loss	6	4.8
Bilateral mixed hearing loss	60	48
Unilateral conductive hearing loss	3	2.4
Unilateral mixed hearing loss	10	8
Single-sided deafness	39	31.2
Other (e.g. one ear conductive, contralateral ear mixed, etc.)	7	5.6

### Evaluated parameters

I. Surgery: type of anaesthesia, duration of surgery, soft tissue reduction, bone polishing, bipolar coagulation use and any surgical problem or difficulty.
II. Healing: any problems with healing and the patients’ subjective feelings concerning pain, numbness and paraesthesia measured by the visual analogue scale – from 0 (absent) to 10 (the strongest possible).
III. State of soft tissue after the processor attachment (activation).

Information on the skin condition and related complaints were recorded on the 10th day after the surgery, one month after the surgery (ranged between 4 and 6 weeks, on the day of processor activation) and 3 months after the implantation. Additionally, in the case of a problem in the operated area, all the patients had a possibility to come for a supplementary visit at any time after the surgery.

## Results

### Surgery

During the surgery, 96% of patients received local anaesthesia and 4% general (5 cases: 3 first surgeries, 1 teenager and 1 patient with epilepsy). The system was implanted on the right side in 58 cases (46.8%) and on the left side in 67 (53.2%). The mean surgery time (from local anaesthesia to final dressing) was 42 min (range from 25 to 70) and was dependent on the need for soft tissue reduction and bone polishing (no reduction and no polishing – 39.4 min, reduction and polishing – 46.5, only reduction – 41.7, only polishing – 46.5). A typical C-shaped incision was performed in all the cases. There was a need for soft tissue reduction in 43.2% of patients, bone polishing in 23.2% and bipolar coagulation use in all the cases. Various surgery related problems were observed in 12.1% of patients (15 cases): (1) bleeding from an emissary vein (3 cases); (2) intensive cortical bleeding from the bone (3 cases); (3) bone thickness <3 mm at the primary implant site requiring new repositioning without any additional skin incisions (3 cases); (4) very soft bone at the primary implant site (5 cases, a new hole was drilled in three of them); (5) pneumatic air cell at the primary implant site, which required a new hole to be drilled (1 case).

### Healing

Healing was uneventful in 92.8% of cases. 7.2% of patients (cases with soft tissue reduction) were diagnosed with haematoma on the day after the surgery, which was successfully treated by suction and compression during the following days. On the first postoperative visit (10 days after the surgery), pain was reported by 48%, numbness by 84% and paraesthesia by 15.2% of patients. During the second follow-up visit (4–6 weeks), pain was present in 18.4%, numbness in 60%, and paraesthesia in 11.2%. Three months after the surgery, pain was reported by 2.4%, numbness by 17.6%, and paraesthesia by 1.6%. The mean intensity of pain, numbness and paraesthesia during the first, second and third visit is presented in Table 2.

**Table 2:** The mean intensity of pain, numbness and paraesthesia measured by visual analogue scale (from 0 to 10) in the group of implanted patients.

	First visit (10 days after surgery)	Second visit (4–6 weeks after surgery)	Third visit (3 months after surgery)
Pain	1.02	0.27	0.05
Numbness	3.04	1.35	0.27
Paraesthesia	0.48	0.14	0.02

### State of soft tissue after the processor attachment

After the processor attachment, no serious problems were observed in the patients during follow-up visits. However, in 9.6% of patients, mild redness and/or mild pain over the magnet was observed. In all these cases, the reduction in the magnet strength or limitation in the daily usage of the processor was advised, which led to problem-solving.

## Discussion

Devices which use bone conduction have been successfully implanted for many years and nowadays various systems with percutaneous abutment or magnetic attraction are used all over the world, along with different surgical techniques. The observation presented in the study concerns implantation of Baha® Attract – the device which was introduced in 2013, and, to the best of our knowledge, it is the largest series of patients operated on in a single department described so far.

In contrast to Işeri et al., Briggs et al. and Hougaard et al., who conducted surgery exclusively or mainly under general anaesthesia,^7^, ^8^, ^9^ 96% of the patients in our study were operated under local anaesthesia. As the postoperative data shows, this approach proved to be safe and suitable for the majority of adult patients. The mean surgery time in our study was 42 min, and was similar to those described in the literature, which ranged between 37 and 57 min.^7^, ^8^, ^9^, ^10^, ^11^ All our surgeries were performed with a typical incision consistent with Cochlear Baha® Attract Surgical Quick Guide, however, in the literature, some modifications of this incision or quite different linear incision are described.^12^, ^13^ The soft tissue reduction in our study was necessary in 43.2% of patients, and was significantly higher in comparison to the already reported, which ranged between 11.1% and 31.2%.^8^, ^10^, ^11^ The difference can be explained only by the individual differences in skin and subcutaneous tissue thickness. The study presented by Wróbel et al., utilising ultrasound examination of the skin thickness in the retroauricular region, revealed positive correlation between skin thickness and weight and the body mass index (BMI), but only to the limited extent.^14^ In the case of the Baha® Attract system surgery, the decision on soft tissue reduction depends on the results of direct soft tissue measurements with the use of a dedicated tool. Bone polishing in our group was done in 23.2%, and was lower than the previously reported (30–41.6%).^7^, ^11^ In our opinion, it is the effect of experience which allows to choose the optimal place for implant and the optimal angle of drilling. The study revealed other surgical problems in 12.1% of patients, which are reported in the literature to a very limited extent. These includes bleeding, bone morphology (condition) and bone thickness. Only Hougaard et al. described the use of a 3 mm implant in cases when the dura was exposed after 3 mm drilling.^9^

No major postoperative complications were observed, and healing was uneventful in most of the cases. However, in 7.2% of patients, a small haematoma was observed on the day after the surgery. Similarly, previous studies report postoperative haematoma or seroma, successfully treated by aspiration and dressing.^7^, ^10^, ^15^

Pain and numbness observed in our study are typical after the Baha® Attract surgery, and were reported previously. Carr et al. observed mild pain in 30% and numbness in 80% of the implanted patients.^11^ Dimitriadis et al. concluded that skin numbness superior to the C-shaped incision is a common complication, but reinnervation occurs in time, and the numb patch decreases in size.^12^ Briggs et al. found numbness at the time of initial fitting (the 4th week) in 62.9%, but after the 3rd month they were still present only in 25.9%.^8^

Despite uneventful postoperative recovery and skin healing, problems at the implant side can occur after processor attachment. They are usually caused by a too strong external magnet. Currently, there are six magnet strengths available on the market. The choice of the magnet strength for the patient is subjective, dependent on stability of attraction, comfort of the patient and personal experience of the surgeon. Hence, there is a need to change it over the time in some cases. Tenderness, redness or pain at the implant side requiring reduction in the magnet strength or limitation in the daily usage were described in the literature.^8^, ^10^, ^13^, ^15^ Reddy-Kolanu et al. observed tenderness at the side of the implant in 17.6%,^13^ Dimitriadis et al. skin tenderness and redness in 3.8%,^3^ Iseri et al. pain in 18.7% and redness with pain in 6.2%,^10^ and Briggs et al. mild erythema in 14.8%.^8^ In most of these studies the follow-up after Baha® Attract implantation was shorter than 1 year, so the evaluation of the long-term effect of magnetic attraction on the soft tissue condition requires further studies. Additionally, severe infection at the implant side or skin necrosis over the magnet, leading to the need of magnet removal and further conversion to connect system after wound healing, were described in individual cases.^13^, ^16^, ^17^ All these serious complications were observed in a short time after processor attachment (initial fitting period) and were caused by too strong external magnet. It indicates that the proper magnet strength and suitable care in the first weeks of using the device are crucial to avoid soft tissue problems.

In our study we observed mild redness and/or mild pain over the magnet after processor activation in 9.6%, which dissolved after typical treatment, and no serious complications were found. However, the observation time is short and there is a need of further follow-up and recording of any adverse effects of the magnets compression.

## Conclusion

Implantation of the Baha® Attract system is an easy and safe procedure. It can be performed under local anaesthesia in adults. There are no major surgical problems or complications, and in most patients, healing process is very efficient. Postoperative pain is usually mild and gradually decreases in the following months. Numbness in an operated area is frequent, but as reinnervation occurs in time, the numb patch decreases in size and finally completely disappears in most cases.

## Conflicts of interest

The authors declare no conflicts of interest.
